# Multiproxy study reveals equality in the deposition of flaked lithic grave goods from the Baltic Stone Age cemetery Zvejnieki (Latvia)

**DOI:** 10.1371/journal.pone.0330623

**Published:** 2025-09-10

**Authors:** Anđa Petrović, Jessica Bates, Aija Macāne, Ilga Zagorska, Mark Edmonds, Kerkko Nordqvist, Aimée Little

**Affiliations:** 1 Department of Archaeology, Faculty of Philosophy, University of Belgrade, Belgrade, Serbia; 2 Centre for Artefacts and Materials Analysis, Department of Archaeology, University of York, York, United Kingdom; 3 Department of Cultures, University of Helsinki, Helsinki, Finland; 4 Institute of Latvian History, University of Latvia, Riga, Latvia; 5 Helsinki Collegium for Advanced Studies, University of Helsinki, Helsinki, Finland; 6 Chair of Laboratory Archaeology, University of Tartu, Tartu, Estonia; Tel Aviv university, ISRAEL

## Abstract

The *Stone Dead Project* carried out analysis of the flaked lithic assemblages from burial contexts at Zvejnieki cemetery, Latvia. Zvejnieki (c. 7500−2500 cal. BC) represents one of the largest Stone Age burial grounds in Europe, consisting of over 330 burials containing 350 individuals. Using a multiproxy approach, combining geological, technological, functional, spatial and depositional context information, we compare the biographies of lithic grave goods with biographical information (age, sex) of the humans they accompany. Results show gender equality in lithic offerings whilst children are the age group most frequently given lithic grave goods. Certain typologies appear to have carried particular significance and were possibly made, and sometimes broken, as part of funerary rituals. The implications of these and other findings are discussed and placed within their broader archaeological context.

## Introduction

**Fig 1:**
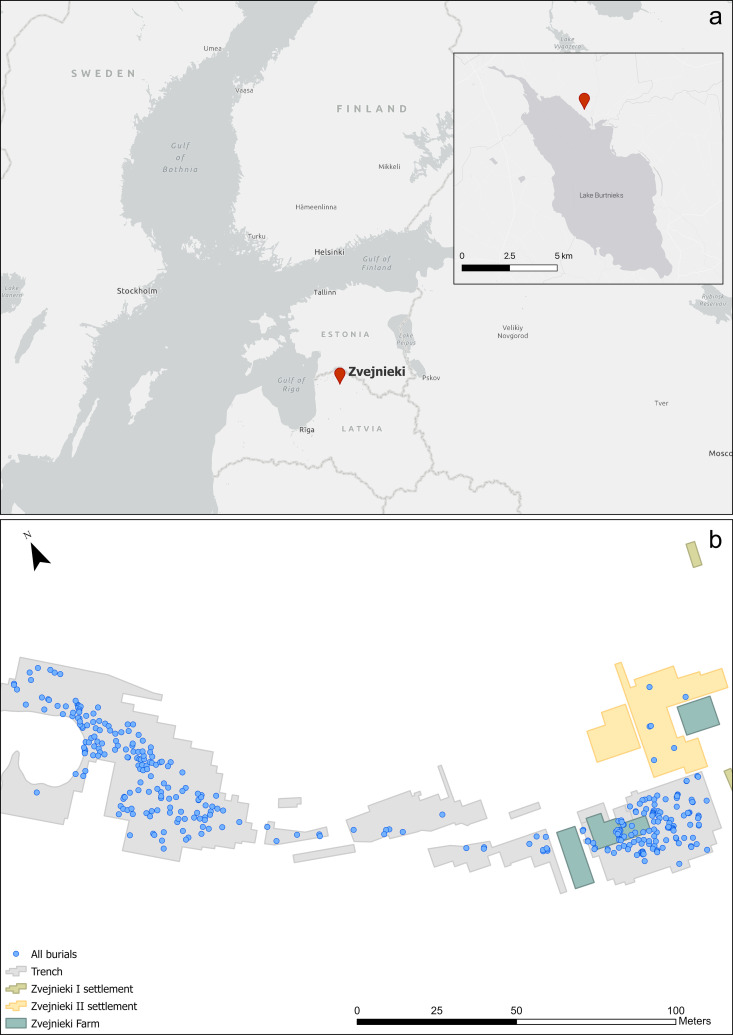
Location of the Zvejnieki burial site and spatial distribution of burials. Location of the site on Lake Burtnieks, northern Latvia (a) Basemap © Estonian Environment Agency, Estonian Land Board, National Land Survey of Finland, Esri, TomTom, Garmin, FAO, NOAA, USGS [4]; Spatial distribution of the 330 known burials at Zvejnieki (b). Map image was produced by the authors and is the intellectual property of Esri and is used herein under license. Copyright © 2025 Esri and its licensors. All rights reserved.

The overarching aim of this study is to shed new light on the reasons why Europe’s Stone Age (Mesolithic and Neolithic) hunter-gatherers gave stone tools to their dead. Achieving this aim, we argue, will help bridge a significant gap in Stone Age mortuary studies, which has historically seen a divide between the level of analytical and theoretical focus given to different types of grave goods [1–3]. To date, personal ornaments have received far greater analytical attention and are typically regarded as symbolic – entangled with different aspects of social identity. In contrast, lithics in mortuary contexts have received far less analytical focus. To help rectify this situation and develop a deeper understanding of this understudied category of grave goods, Zvejnieki, located in northern Latvia (Fig 1), was chosen as the case study site to carry out in depth research on lithics from burial contexts.

Zvejnieki represents one of the largest Stone Age burial sites in Europe: spanning more than 5000 years, from 7500 to 2500 cal. BC, with a small number of more recent burials [5,6]. Excavated over several seasons mostly between 1964–1978 and 2005–2009, Zvejnieki’s long chronological sequence and relatively well-documented archive of human skeletal remains and associated artefacts provides crucial insights on the death rituals of Stone Age communities in Latvia and beyond. Access to the Zvejnieki cemetery lithic inventory, made possible by the Latvian National Museum of History, enables an exploration of variation in the deposition of lithics, of different typologies and geologies, across time and space (see also [1]). To address our aim, we identified a series of interlinked objectives, which can be summarised as follows:

A. To investigate whether broad spatial and chronological patterns in the deposition of flaked lithic grave goods can be identified at an intra-site scale.
B. To carry out microwear analysis on lithic artefacts, to understand the character of their use and treatment over the course of their lives.
C. To assess whether variation in typologies, geologies and wear traces on lithic artefacts can be identified in relation to an individual’s sex, age, and other available biographical information.

### Zvejnieki in context

The Zvejnieki archaeological complex, consisting of a cemetery and a large settlement area, is located on a former island (a drumlin) on Lake Burtnieks, northern Latvia (Fig 1a). The site was discovered during gravel extraction in 1964 and extensively excavated during the 1960s and 1970s, with new field campaigns conducted in the early 2000s [5,7,8]. In total, 330 graves have been excavated so far, with at least 350 individuals recorded ( [5,9]; Fig 1b; see also S1 File). However, it is likely that more burials exist, yet to be excavated, in both the cemetery and settlement areas. The settlement is located adjacent to the cemetery but is yet to be published. Additionally, loose human bones recovered from both the cemetery and settlement suggest the total MNI is considerably higher than published estimates [10].

The Zvejnieki archaeological complex was used during the Mesolithic and Neolithic, which in local periodization are both characterized by the hunter-fisher-gatherer lifestyle. Existing radiocarbon dates suggest that the area was used for settlement from the 9th millennium BC onwards [8,11]. The main period of cemetery use is dated approximately 7500–2500 cal. BC, with occasional burials up until the historical period [6,12]. Prehistoric burials are distributed over a 200m long area with varying density. Site excavators ( [5]; see also [6]) have suggested that there is a general gradual spatial transition from Mesolithic to Neolithic burials when moving from NW to SE. However, radiocarbon dating (^14^C) suggests that patterning through time is rather more complex, even if the general trend holds at a broad level [9,12].

Inhumation predominates at Zvejnieki. Simple flat-earth graves are furnished with occasional stone settings and fills of organic-rich material from the adjacent settlement site [5,8,9], as well as red ochre, found in almost half of the burials (n = 173; see [5,13]). Approximately one-third of the buried individuals were given grave goods, most often made of bone, antler and teeth, but also minerals such as amber and clay. Based on previously published and available data [7,8], out of the total number of 350 individuals, regardless of context type, 146 were associated with artefacts found either in primary or secondary contexts (see below). In a more recent study, involving a complete overview of original archaeological documentation, this number increased to 154, of which 115 are individuals with artefacts from secure (i.e., primary) contexts [9], (see S1 File). Our study of published and unpublished excavation reports, and the museum archive, has revealed a total of 33 burials containing lithics at Zvejnieki and n = 158 individual flaked lithic artefacts from primary burial deposits. All 158 artefacts were analysed for basic geological information, technology/typological attributes, and archaeological wear traces. This data provides the basis for the research presented here (see S1 File).

## Materials and methods

### Inclusivity in global research

The primary analysis for this study was carried out at the Latvian National Museum of History, with permission to analyse the Zvejnieki lithic collections given by the administration and curatorial staff. Ethics approval was provided by the Department of Archaeology, University of York, Ethics Committee. No permits were required for the described study, however, permits were sought/given for the use of images. Our study has complied with all relevant regulations. Additional information regarding the ethical, cultural, and scientific considerations specific to inclusivity in global research is included in the Supporting Information (S2 File).

### Limitations and challenges

Given that most of the excavations at Zvejnieki were carried out decades ago, the archive is not without limitations. Excavations were undertaken using methodologies and standards of their time, involving no sieving and the use of large recovery units. Whilst we have done our best to circumnavigate some of these challenges, several issues, and thus biasing factors, likely remain. Such limitations, we argue, need to be openly discussed and factored into the interpretation of the Zvejnieki assemblages – here and in any future outputs derived from the site. We start by setting out the four main challenges relating to the lithic archive, with the issues raised often impacting on other forms of material culture.

#### Excavation methods.

Considering the excavation methods used and the composition of the settlement and cemetery finds, it can be assumed that the debris was not completely collected. This applies to lithic artefacts but also probably other classes of material, such as unworked bone. Moreover, given the research focus at the time, there was likely a bias towards retaining “exotic”, unusual and/or formal objects compared to simple forms made from locally available materials.

#### Documentation.

Discrepancies are occasionally found between the original excavation reports, the main publications [5,7,8], and what is physically present in the museum collections. Original unpublished excavation reports and documentation, as well as all published data and illustrations, were consulted when determining the find context of each artefact. From a techno-typological point of view, our data is based on in-person observations of the materials, and thus may differ on occasion from previous publications.

#### Infilling of graves with settlement soil.

More challenging issues arise when determining the context of individual lithic finds, namely whether they formed part of a primary deposit or were part of the grave fill (referred to here as **SD** for **Secondary Deposits**). These challenges were already recognised during the excavations [7,8]. Some lithics form part of the primary grave assemblage; we have labelled these **PD** for **Primary Deposits** in that they were found in direct association with the human remains. Also included are lithics found in structured deposits within the graves that were spatially, and likely, temporally associated with burials (labelled **OPD** for **Other Primary Deposit**) (see Table 1), which have previously been referred to as discrete “votive deposits” [7]. Excluded from our analyses were lithics contained within the burial fill (or in some cases, the soil spread under the body), which was taken from the nearby settlement. These lithics cannot be classified as intentional grave offerings and instead are considered secondary deposits. Similarly, artefacts labelled as coming from “the vicinity” of a burial have not been included here.

**Table 1:** Description of Zvejnieki cemetery lithic contexts. Description of Zvejnieki cemetery lithic contexts after Zagorskis [7]; Larsson et al. [8] with Stone Dead Project’s categorisation and abbreviation, see S1 File.

Context description	Simplified classification	Stone Dead Project abbreviation
Lithics are deposited directly into the grave and associated with the body. Sometimes these are clearly connected with specific body parts, other times not.	Primary Deposit	PD
Lithics are deposited within the grave, near the body in a separate structured deposition but associated with the primary mortuary deposits.	Other Primary Deposit	OPD

#### Intra-burial spatial associations.

The primary literature varies in the level of detail provided for associations of body-parts and artefacts [5,7,8]. For the purposes of this study, we have relied upon recorded associations with main body parts, e.g., head, torso, legs, feet and hands. ‘Unknown’ is used when no information is available, which was unfortunately the case on several occasions, generally due to disturbance.

### Sampling

With the exception of cores (n = 13), all flint artefacts found in primary contexts were selected for functional analysis. None of the cores were modified into core tools or had any macrowear suggesting their use as a tool. The five axes found in association with Zvejnieki burials have been published elsewhere [1]. Whetstones and other types of coarse stone tools have also been excluded from our analysis because they present very different geological and technological considerations and are therefore not an appropriate comparative dataset for the flaked lithic burial assemblages.

### Database design and access

The Stone Dead Project database was built using a Google spreadsheet and is hosted by the Archaeological Data Service. More specific details concerning the design of the database and classifications used are provided in S1 File.

### Spatial and contextual analysis

Burials **1**–**302** were excavated over several field seasons between 1964 and 1971, with a further six burials discovered during the excavation of the Zvejnieki II settlement in 1972–1977 [5,7]. More recent excavations of the cemetery took place from 2005 to 2009. Numbering of these burials (see [8]) follows on from Zagorskis [7], commencing at **309** and finishing at **330** (Table 2). In general, each individual, even when forming part of a double or collective burial, was given a unique burial number identifier. Exceptions include the infants who were only identified during post-excavation osteological analysis and are separated by the identifier “a” (e.g., 85–85a; see [5]) as well as the collective burial **312**, which is numbered as **312a-d** [8]. In a few cases, the identifier “a” was not provided, with bones from two individuals identified under the same grave number (e.g., **119**, **138**), bringing the total number of individuals to 350.

**Table 2:** Burials, numbered 1-330, their year of excavation and relevant publication.

Burial numbers	Excavation years	Key references
1-308	1964, 1965, 1966, 1968, 1970 and 1971, 1972, 1975, 1977	Zagorskis 1987, 2004
309-330	2005, 2006, 2007, 2009	Larsson 2010; Larsson et al. 2017

### Geographical information system

Spatial coordinates and/or grid square information on the specific location of lithics within each burial were not always recorded during the earlier excavations but were meticulously recorded during 2005–2009 field campaigns. Spatial coordinates of trench and burial locations, previously published by the Stone Dead Project (see [1]), used trench layouts from Zagorskis [5] and Larsson et al. [8]. These coordinates were used to visualise all burials and were joined with the lithics database to facilitate spatial analysis. Each burial was digitised as a single point using GIS (Geographical Information System) software ArcGIS Pro 3.2.2. If a burial contained multiple lithics, these could not be visualised as separate points. To identify spatial patterns in the data, colour was used to differentiate quantities of each tool type associated with individual burials.

### Lithic analysis

**Fig 2:**
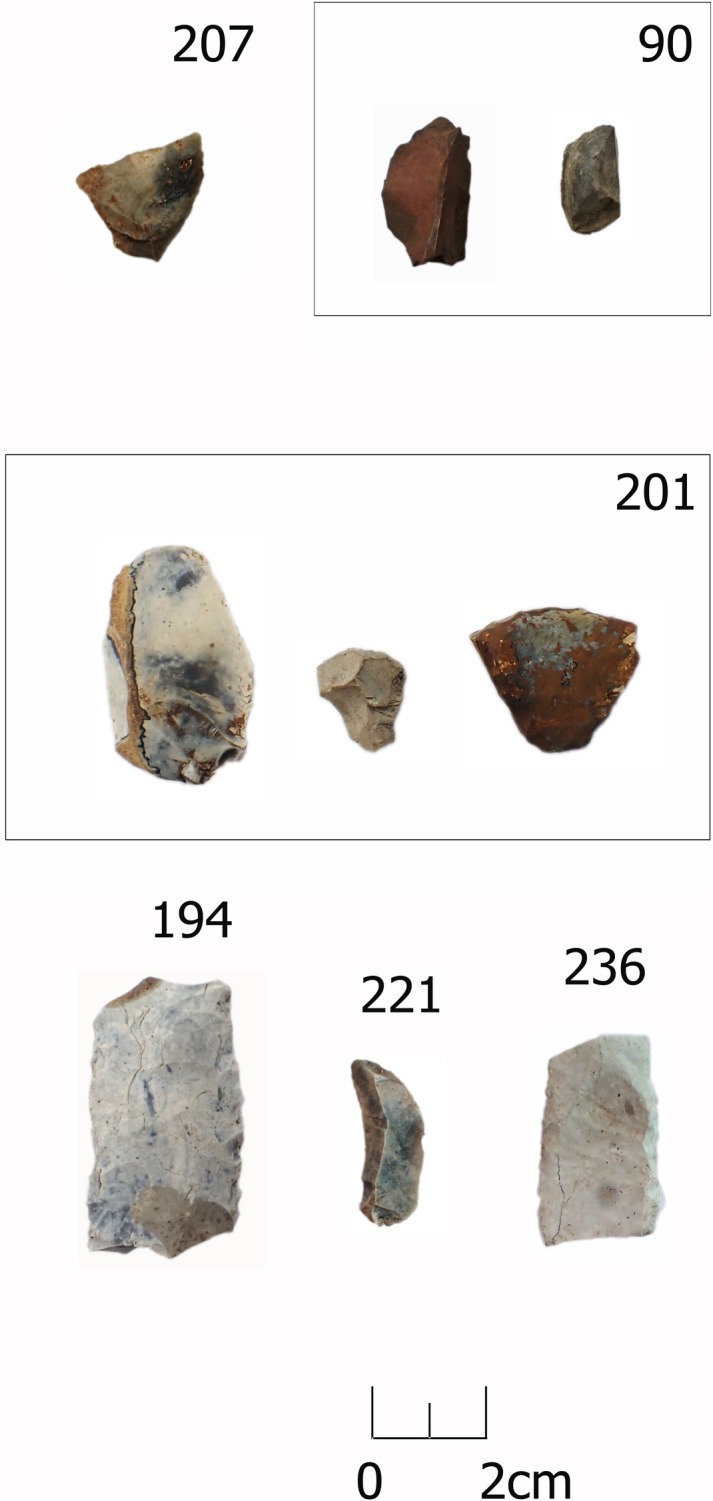
Non-patinated, patinated and thermal stress lithic artefacts. First row (non-patinated) – burial 207 (sample 145, VI93:420a), burial 90 (samples 34, 35, both under the same museum no. VI93:117) affected by thermal stress; Second and third row – Artefacts partially and completely covered with patina and blue film representing the initial stage of patina formation, burial 201 (samples 134–136, VI93:391–393); burial 194 (sample 115, VI93:374), burial 221, (sample 242, VI93:374), burial 236 (sample 251, VI93:678). Artefacts are part of the Zvejnieki cemetery collection (VI93) stored in the Latvian National Museum of History*.*

Flaked lithic artefacts of different raw materials, though primarily flint, underwent geological assessment, technological and functional (microwear) analysis. The condition of the lithics was generally quite poor, in large part due to patination and to the varying quality of the flint itself (Fig 2). Below we outline the primary methods employed, acknowledging key challenges and limitations, when present (see also S1 File).

#### Geology.

Broad geological classifications – flint, quartz/quartzite, slate – were used and based on visual observations (see S1 File). Quartz/quartzite and slate, few of which were found, may have been sourced locally in glacial deposits (tills). Most of the analysed material is classified as flint, mostly imported material from Cretaceous or Carboniferous deposits in neighbouring territories (Lithuania, Belarus, western Russia), though lower-quality, locally available Silurian flint is also present (see [14,15]). Although it is common practice in local archaeological tradition to determine the origin of flint by colour, we have used this technique with caution because colour determination is not entirely reliable due to discoloration and patina (see Fig 2).

#### Technological analysis.

**Fig 3:**
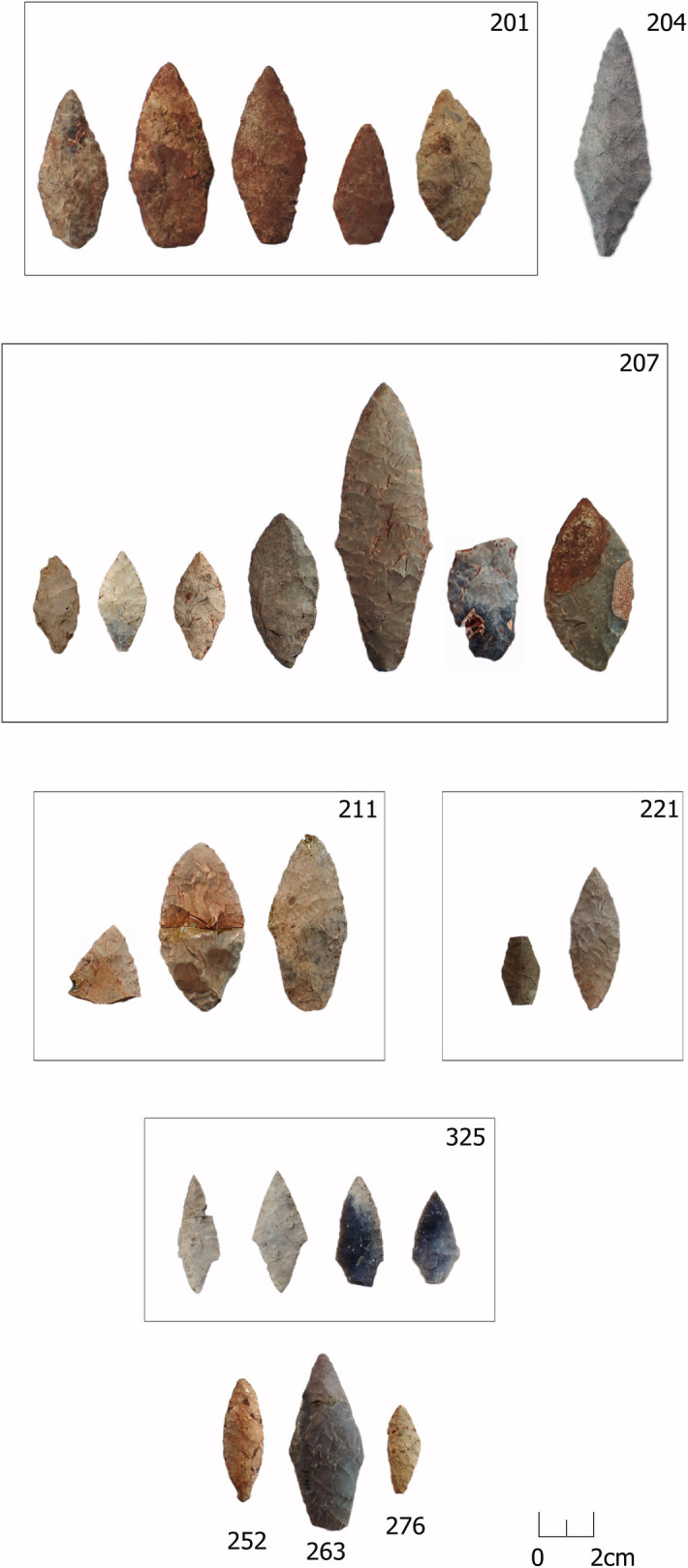
All bifacial points from PDs and OPDs. Burial 201 – samples 137–14 (VI93:386–390); burial 204 – sample 142 (VI93:406); burial 207 – samples 146 (VI93:420), 159 (VI93:423), 165 (VI93:432), 162 (VI93:427), 166 (VI93:442), 167 (VI93:444), 170 (VI93:451); burial 211 – samples 192 (VI93:475), 193 (VI93:473), 194 (VI93:474); burial 221 – samples 243 (VI93:599), 244 (VI93:600); burial 325 – samples 299–302 (museum numbers not assigned); burial 252 – sample 272 (VI93:702); burial 263 – sample 275 (VI93:719); burial 276 – sample 290 (VI93:746). Artefacts are part of the Zvejnieki cemetery collection (VI93) stored in the Latvian National Museum of History*.*

Broad technological and typological categories were recorded, including reduction sequence. Typologies distinguish flakes, blades, knives, scrapers and bifacial points (Fig 3), the latter term commonly used in the eastern Baltic area for bifacially flaked artefacts, which in older literature may also appear as arrowheads, spearheads, and even knives (e.g., [5,7]). Although scrapers and knives are effectively a retouched blade or flake, we have opted to record the secondary/formal tool classification rather than the primary one. If a flake or a blade has evidence of retouch, this is recorded in a separate column. Similarly, bifacial points are, as might be expected, recorded as retouched/worked.

#### Functional analysis.

Microwear analysis was undertaken at the Latvian National Museum of History (Riga) over a period of several months during 2021−2022, with final checks completed in 2024. Analysis was carried out when the condition of flint was suitable (patination was a limiting factor). Lithics from Zvejnieki burials were washed during the excavations and subsequently heavily curated. Nonetheless, all the samples were analysed with an aim to recover traces of any recalcitrant residues like ochre and tar. Both low- and high-power approaches [16–19] were employed. A stereo microscope BRESSER Science ETD-201 (magnification 8x-50x) was used for macro-observations (courtesy of V. Bērziņš, University of Latvia). An Olympus BX53MRF (x5, x10, x20 oculars) metallographic microscope with an Olympus digital camera SC180, equipped with the Stream Basic software, was imported from the UK (University of York) and used for high power analysis. Additionally, photos were processed with Helicon Focus, a software for processing digital images, when needed.

### Other grave goods and red ochre

Other types of lithic (coarse and polished stone) and non-lithic (osseous, amber, etc.) grave goods were not studied; only their presence/absence is recorded as “other grave goods” (See S1 File). The use of ochre (an iron-rich mineral pigment) in the burial was similarly recorded as present/absent; another column records the presence of ochre staining on the analysed individual lithics. Information on other grave goods and ochre was drawn principally from Zagorskis [5,7], Larsson et al. [8], and Macāne [9].

### Human remains

#### Sex and age.

Sex and age identifications for the skeletons excavated in the 1960s and 1970s took place at the time of excavation with advice from specialists. This data is presented in the original publication by Zagorskis [5]. However, and as explained in an editorial note [7], information was updated in the English edition based on analyses conducted by anthropologists [7,20–22]. In cases where skeletal remains no longer survived, the original determinations were retained. Sex determinations in the *Stone Dead Project* database (see S1 File) are based on the osteoarchaeological report in Zagorskis [7], with reference to the original report when discrepancies existed between the two. Data for the skeletons excavated in the 2000s is adopted from Larsson et al. [8] and Nilsson Stutz [23], Nilsson Stutz & Larsson [24]. Since different age classifications were used by Zagorskis [7] and Larsson et al. [8], the Stone Dead Project employs simplified age categories to avoid confusion (Table 3).

**Table 3:** Age group categories.

Zagorskis 2004	Nilsson Stutz 2010, Nilsson Stutz & Larsson 2016; Larsson et al. 2017	Stone Dead Project simplified classification (see also S1 File)
Infans I, 0–7 years	newborn, child	younger child
Infans II, 7–14 years	n/a	older child
Juvenis, 14–19 years	juvenile	younger adult
Adultus, 20–40 years	adult, 25–40 years	adult
Maturis, 40–60 years Senilis, over 60 years	n/a	older adult

Age group categories used in Zagorskis [7], Nilsson Stutz [23], Nilsson Stutz and Larsson [24], Larsson et al. [8], and the simplified system used by the Stone Dead Project.

Genetic sexing was undertaken primarily using petrous bone for a limited number of individuals [25,26]. Only in three cases, does the DNA sexing contradict osteological sexing (burials **121, 137, 221**; only **221** contained lithics). The sex of five individuals with lithics is determined genetically, including four children (burials **67, 99, 128, 207**; the fifth sexed individual, **221**). These children are the only ones we have allocated a sex due to the well-known issues with sexing children based on osteological identifications [27–29]. A few of the more recently excavated burials, in a poor state of preservation, are indeterminate both in terms of age and sex [23,24].

### Dating of human skeletal remains and associated material culture

Radiocarbon dates are only available for a limited number of burials (e.g., [6,12,26,30–33]). In cases where an individual with lithics but without a radiocarbon date is part of a double or collective burial in which dates for other individual(s) or grave goods are available, the latter were used as a proxy date for the entire burial (individuals **90**, **207**, **317**). Material culture and other diagnostic burial customs were used as chronological criteria for burials without radiocarbon dates. A small number of burials lacked dates and diagnostic features; these were classified “unknown”.

Since forager diets in general contain high amounts of aquatic components, the radiocarbon dates are affected by dietary reservoir effect. Reservoir corrections have been applied on some of the dates (see [12,32,33]), but the consequent broad time frames may still contradict the typo-chronological ages based on material culture. In these cases, the material culture was given precedence. Given this, and the long temporal depth of the cemetery, ‘millennium’ was seen as the most suitable comparative temporal reference unit for the purpose of this study.

## Results

### Temporal and spatial analysis of primary lithic deposits

In total, 31 burials (9% of all burials) contain primary deposit lithics, and 3 burials (1% of all burials) contain lithics in OPD; only burial **207** contained lithics both in PD and OPD (Table 4).

**Table 4:** Burials and associated types of deposits.

Burial Numbers	Type of deposits
3; 12; 24; 57; 67; 82; 85; 90; 95; 99; 114; 128; 144; 153; 192; 194; 201; 204; **207**; 221; 223; 236; 242; 263; 264; 276; 277; 282; 317; 320; 325	Primary
**207**; 211; 252	Other Primary

Bold indicates the only burial (207) with lithics in PD and OPD.

**Fig 4:**
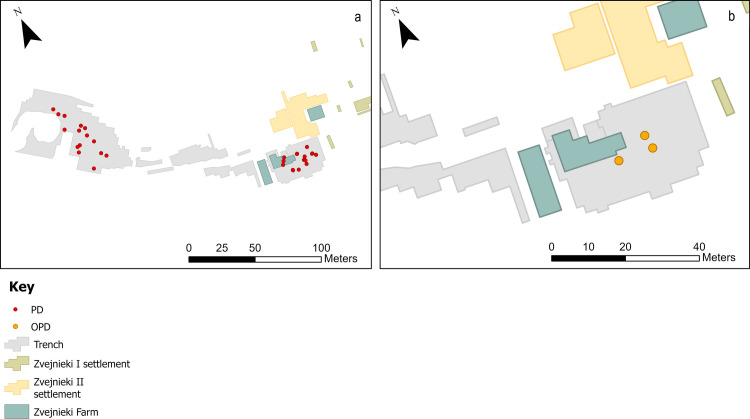
Map showing the distribution of burials with PD (a) and OPD (b) lithics.

**Fig 5:**
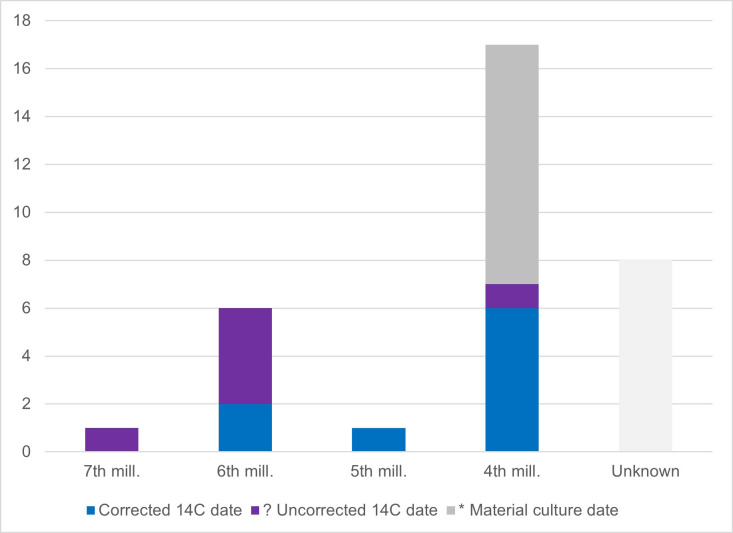
Bar chart showing number of PD and OPD lithic burials associated with each millennia category.

**Fig 6:**
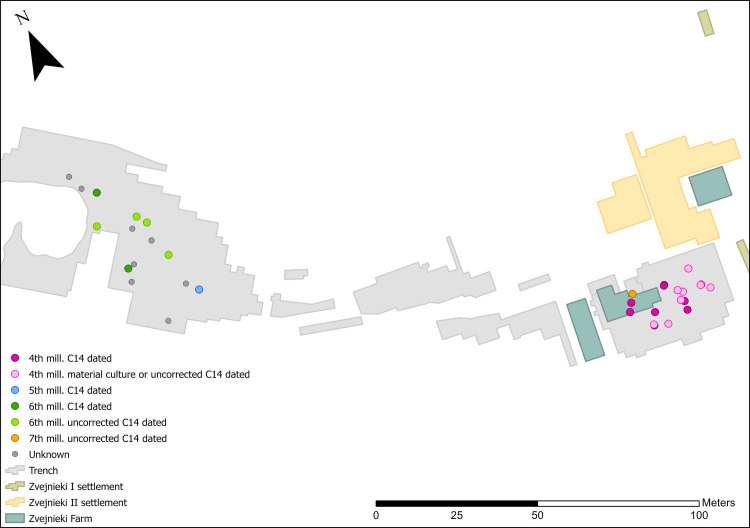
Spatial distribution of millennia dates for PD and OPD burials.

Primary deposits with lithics are found relatively evenly spatially distributed in both the NW and SE parts of the cemetery. There is a notable absence in the central part (Fig 4a-b GIS map): reflecting an overall much lower number of burials in this area (see Fig 1b). If the quantity of finds is considered instead of the number of burial contexts, the situation changes: the 14 PDs in the NW contain a total of 24 items, while the 19 burials with PDs/OPDs in the SE contain 134 pieces. Spatial distribution of lithic PD and OPD, with millennium dates assigned (Fig 5), clearly shows the NW versus SE divide, with NW burials dating to the earlier phases – 6th and 5th millennium BC, and the SE of the site containing later burial activity in the 4th millennium BC, bar one 7th millennium BC outlier (burial 320) (Fig 6).

With one exception (burial 320), all burials located to the SE date to the 4th millennia BC, compared to those to the NW which date to either 6th or 5th millennia BC.

### Sex/gender

#### Sex all individuals.

Of the 350 individuals recorded at Zvejnieki, n = 133 (38%) are biologically male or possibly male; n = 58 are biologically female or possibly female (16%) and 159 (46%) are indeterminate. This latter category is inflated due to the inclusion of children, who, bar those individuals sexed using DNA methods (n = 7), cannot be sexed osteologically (see Table 5).

**Table 5:** Quantities of different sex categories of all 350 individuals from Zvejnieki and presence of artefacts for all individuals.

Sex category	All individuals (n = 350)	Individuals found with artefacts (n = 146)
Female (includes Female?)	50	18
Female (DNA sexed)	8	5
**Combined female**	**58**	**23**
Male (includes Male?)	118	50
Male (DNA sexed)	15	8
**Combined male**	**133**	**58**
Child indeterminate	92	47
Adult indeterminate	63	18
Indeterminate	4	0
**Combined indeterminate**	**159**	**65**

#### Sex of individuals with lithics in primary deposits and other primary deposits.

When lithics in PD and OPD are looked at as a percentage of each sex (Table 6) there is no real difference between their frequency of occurrence. In fact, contrary to common narratives which emphasise the relationship between lithics and male burials (e.g., see [3] for discussion) females are slightly higher at 12% compared to 11% of all males.

**Table 6:** Sex categories of individuals with PD and/or OPD lithics.

Sex category	Individuals found with PD and OPD lithics (n = 33)	Percentage of sexed individuals with PD and/or OPD lithics
Female (includes Female?)	4	8%
Female (DNA sexed)	3	37.5%
**Combined female**	**7**	**12%**
Male (includes Male?)	13	11%
Male (DNA sexed)	2	13%
**Combined male**	**15**	**11%**

Percentages represent the total number of individuals associated with each sex category (see Table 5), divided by those containing PD and/or OPD lithics.

#### Age of all excavated individuals at Zvejnieki.

The largest percentage of the 350 individuals buried at Zvejnieki are adults (Table 7). In broad terms, adults (including those indeterminately classified as ‘adult/older adult’) make up 49% (n = 173) of the total buried population; 15% (n = 52) are older adults; 4% are younger adults (including those indeterminately classified as “younger adult/adult”); 30% (*n* 106) are children (includes younger and older children); a further 1% (n = 4) were so poorly preserved they can only be classified as indeterminate (Table 7).

**Table 7:** Age categories for all 350 individuals and presence of artefacts by age category.

Age category	All individuals (n = 350)	Individuals found with artefacts (n = 146)	Percentage of individuals found with artefacts
**Younger child**	42	21	50%
**Older child**	15	7	46%
**Child**	49	26	53%
**Younger adult**	15	5	33%
**Adult**	173	65	38%
**Older adult**	52	22	42%
**Indeterminate**	4	0	0%

#### Age of individuals with lithics in primary deposits and other primary deposits.

Age results for all individuals with lithics in PD or OPD (n = 33) reveals that the largest percentage by age category is, in fact, younger children (19%), followed by older adults (17%) (see Table 8). Somewhat surprisingly, the age category “adult” is one of the lowest (7%). The same proportions of older children and younger adults (13% respectively) may reflect past societal attitudes which made no distinction between these age classes in death (and possibly also life) – at least, in respect to their association with lithics. However, the quantity of burials in these categories are few, making it difficult to say too much about their significance.

**Table 8:** Age categories of individuals with PD and/or OPD lithics.

Age category	Individuals found with PD and OPD lithics (n = 33)	Percentage of individuals with PD and/or OPD lithics
**Younger child**	8	19%
**Older child**	2	13%
**Child**	0	0%
**Younger adult**	2	13%
**Adult**	12	7%
**Older adult**	9	17%
**Indeterminate**	0	0%

### Lithic analysis

#### Geology.

Most of the analysed items are made from flint (n = 154), with just three pieces of quartz/quartzite and one piece of slate. Flint is usually light beige, brownish-yellow or grey, the only notable exception being the items of white-blueish-grey flint found in some graves. Accepting that there are potential problems in determining the raw material provenance based on colour alone, we tentatively suggest that at least some of the beige and light grey flint is likely raw material of local origin. However, some of the brown and darker grey, as well as the blue-white-grey flint was imported from neighbouring regions. The imported flint – and artefacts made of it – is concentrated in burials dated to the 4th millennium BC [5]). The three quartz artefacts (burials **99**, **264** and **282**) appear to have no particular chronological or spatial distribution.

#### Technology.

Flakes are the most common knapped lithic artefacts (n = 84/53%) found within burials containing PD and OPD; all non-flint lithics (n = 4) are also flakes but are omitted from further discussion here. The flakes are followed by blades (29/18%) and bifacial points (n = 25/16%), scrapers (n = 17/11%) and knives (n = 3/2%) (Table 9).

**Table 9:** Typologies of lithics from Zvejnieki PD and OPD burial contexts.

Typology	Quantity/%
Flakes	84/53%
Blades	29/18%
Bifacial points	25/16%
Scrapers	17/11%
Knives	3/2%
**Total**	**158/100%**

**Fig 7:**
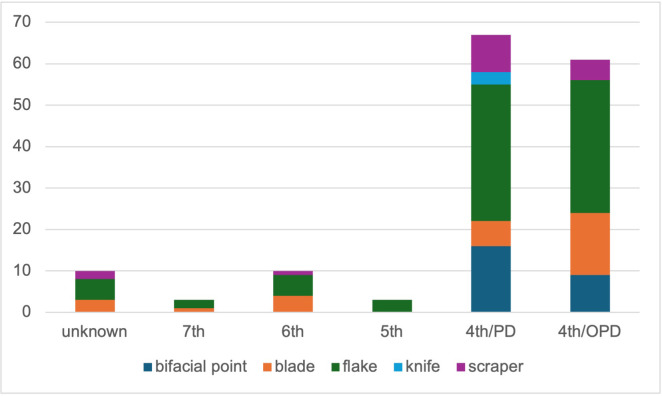
The quantity of main tool types during each millennium.

Knapped lithics appear to have played a more limited role in the burial customs practiced at Zvejnieki before the 4th millennium BC. Of the 154 flints, 128 are associated with the 4^th^ millennium BC burials (14 PD and 3 OPD contexts) and only 26 lithics with other millennia: 3 items (1 PD) with the 5^th^, 10 items (5 PDs) with the 6^th^, and 3 items (1 PD) with the 7^th^ millennium BC; while 10 items (7 PDs) are ‘unknown’ (Fig 7). Thus, the amount of material per millennium is too small to record diachronic trends.

For the 4th millennium BC, PD and OPD are shown separately. All other millennia contain lithics in PD only.

The exclusivity of the lithic finds in burials is further emphasised by the fact that 40% of all flints (61 out of 154 pieces) come from the three OPD, all dating to the 4^th^ millennium BC (48% of the 4^th^ millennium BC lithics). The OPD in burial **211** alone contains 47 artefacts, covering almost a third of all knapped lithic finds in the Zvejnieki cemetery. The three OPDs together include 36% of all bifacial points, 52% of blades, 40% of flakes and 29% of scrapers; only knives are not present. Against this background, it is not surprising that all but three formal artefacts are associated with the 4^th^ millennium BC. This includes 82% of scrapers, all knives as well as all bifacial flint points, whose appearance is commonly dated to the early 4^th^ millennium BC in the eastern Baltic area [14,34]. Bifacial points also differ in their knapping technique (combining percussion and bifacial pressure flaking) from all other lithics in the burials.

Most items in the burials are complete; there are no major differences between PDs and OPDs (74% vs. 80%). Fragmented specimens occur only sporadically in all find categories. Bifaces are the exception to this trend, with around a third occurring as fragments, which suggests a different kind of treatment compared to other lithic artefacts. Blades and flakes are usually intact and show traces of secondary working. The only exceptions are the flakes/blades dated to the 6^th^ millennium BC and “unknown”, where more than half of blades/flakes show traces of secondary working (cf. 7% for material from the 4^th^ millennium BC). However, due to the small amount of material, this may also be incidental.

Of the osteologically and aDNA sexed individuals with lithics in PD and OPD (Table 10), it is interesting to note that no sexed males have scrapers. Bifacial points, whilst they may appear to be more frequently associated with females (n = 12), are in fact concentrated across just three burials; the 8 bifacial points found with males come from six individuals. So, while bifacial points more frequently occur with males, when found with females, they appear in higher concentrations. Similarly, there are more instances of male individuals (n = 4), found with blades compared to females (n = 3); however, when a female grave is furnished with blades, they appear in higher quantities.

**Table 10:** Count of typologies in relation to osteological and aDNA sex categories from PD and OPD.

Sex (DNA*)	bifacial point	blade	flake	knife	scraper	total
F and F* Total *n* 7 individuals	12	21	49	0	8	**90**
M and M* Total *n* 15 individuals	8	5	22	1	0	**36**

In terms of age categories, older children (n = 45*)* and younger adults (n = 50*)* have the highest total count of lithics (Table 11). The most striking observation is that these categories are represented by only four burials. Because high densities of some forms occur in just a small number of burials, a site-wide assessment does not accurately reflect patterns of lithic deposition. For this reason, we have chosen to provide more detailed discussion of typology in relation to specific burials with high densities of lithics (see below).

**Table 11:** Count of typologies in relation to age categories from PD and OPD.

	bifacial point	blade	flake	knife	scraper	total
**younger child** Total *n* 8 individuals	0	3	8	1	0	**12**
**older child** Total *n* 2 individuals	12	0	20	0	13	**45**
**younger adult** Total *n* 2 individuals	3	15	31	0	1	**50**
**adult** Total *n* 12 individuals	4	4	9	2	3	**22**
**older adult** Total *n* 9 individuals	6	7	16	0	0	**29**

#### Microwear and residues.

**Fig 8:**
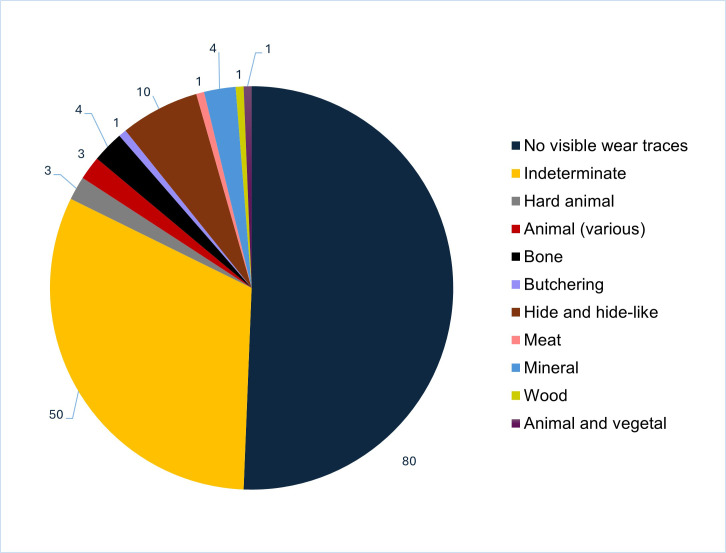
Quantity of the different traces (contact materials) found on lithic artefacts in PD and OPD.

Of the 158 flaked lithic artefacts from secure contexts represented by PDs and OPDs 51% (n = 80) have no visible wear traces, and 32% (n = 50) have non-diagnostic, or as referred to here, indeterminate traces (Fig 8). *No visible wear* traces relates to tools with no evident macro or micro traces. These lithics may never have been used, were possibly used for only a very short period to work a soft contact material or have traces that have not been well preserved. *Indeterminate* refers to lithics with signs of previous use, either through macro or microwear, with traces obscured by post-depositional surface modification (PDSM).

**Fig 9:**
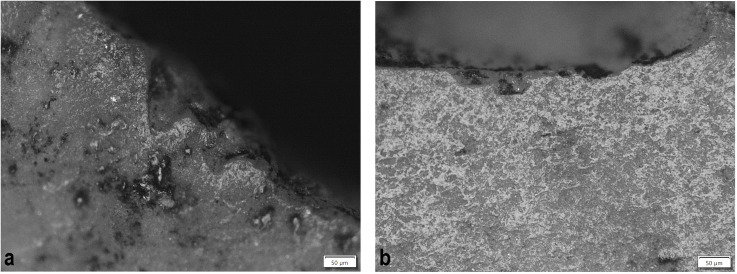
Post-depositional surface modifications on Zvejnieki flint artefacts. Soil sheen on edge and non-diagnostics scars (not developed from use), ventral surface, sample 291 (VI93:772), burial 277 (a); Soil sheen on distal area of the blade, ventral surface, distal area, sample 294 (museum number not assigned), burial 320 (b). All photos taken by metallographic microscope. Artefacts belong to the Zvejnieki cemetery collection (VI93) stored in the Latvian National Museum of History*.*

Out of the represented sample 65% (n = 102) of the artefacts displayed PDSM, sometimes multiple surface alterations. The most common PDSM is characterised by glossy appearance and soil sheen visible as highly reflective areas sometimes covering small (Fig 9a) or larger portions of the object’s surface (Fig 9b). It is unclear what taphonomic factors created the PDSM; material may have laid on the ground, been trampled etc., prior to deposition into the grave, or have become affected by disturbances to the burial environment. Only three flakes had traces of thermal stress, two of them originating from the same context – burial **90**, a younger child (see Fig 2).

Patina is present on 60% (n = 95) of the analysed samples, with white patina the most common type. In some cases, a blue film covering the surface of the artefacts is visible, likely linked to the formative phases of patina development [35]. Microwear information was still retrievable even on a relatively high proportion of patinated pieces. This is demonstrated by a positive assessment of 78 out of 95 flints recorded as having some form of patina.

For the 15 artefacts categorised as *indeterminate* it was possible to analyse macro traces and describe their characteristics, with the following classes of material hardness recognised: hard (2), medium (5), medium to hard (5), soft to medium (2) and soft (1) materials. Whilst these are broad categories, hard materials are usually associated with bone or antler, medium with hide and wood, and soft with meat and plant processing.

**Fig 10:**
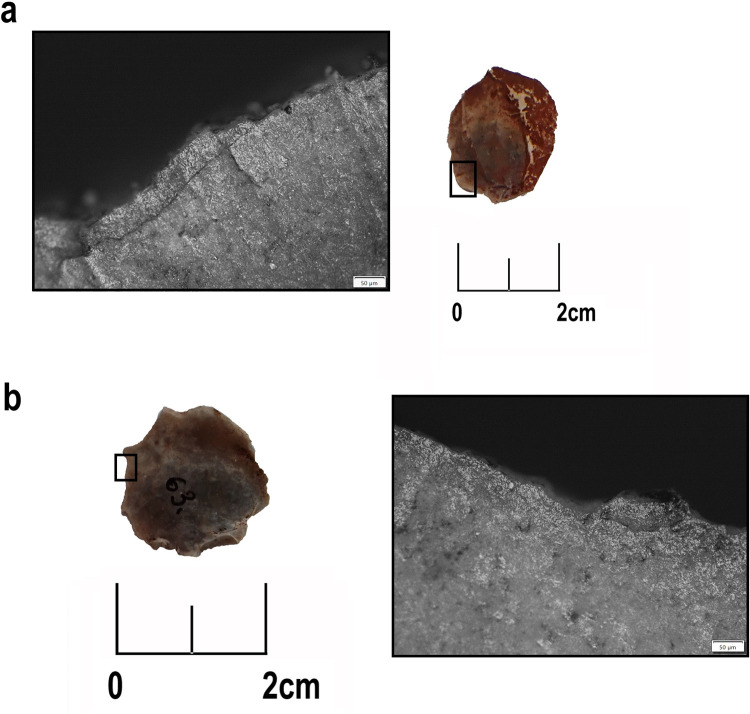
Microwear traces on two flint artefacts. Processing hide, sample 198 (VI93:472), burial 211 (a); Mineral processing, sample 238 (VI93:603), burial 221 (b). All micrographs taken by metallographic microscope. Artefacts are part of the Zvejnieki cemetery collection (VI93) stored in the Latvian National Museum of History in Riga*.*

A greater variety of activities can be observed through the preserved microwear traces. Besides the more identifiable archaeological wear traces, “animal (various)” refers to three pieces with traces which have elements suggesting the processing of some kind of animal material but which cannot be further interpreted. Four lithics displayed bone traces, 10 have traces related to the processing of both fresh and dry hide, while one flake has wood-working traces. Processing of hide has been noted on the majority of diagnostic tools (n = 10) with an occasional distinction between the different phases of work: fresh and dry (Fig 10a). Motion has been observed on around 20% (n = 31) of all analysed artefacts. Cutting, scraping, drilling and a mixed activity of cutting and scraping, defined by the directionality of use-wear scars and micropolish. Cutting is the most frequent action found (n = 22) and has been associated with numerous animal-derived contact materials, such as bone, hide and meat as well as mineral. The combined process of cutting and scraping has been mostly noted in association with working soft to medium material and minerals. Development of wear traces and degree of edge rounding indicates that, overall, tools were mainly used for shorter rather than longer durations of time before deposition.

Tools used for working bone and hard animal materials are associated with nearly all age categories. A similar situation has been noted for hide processing except for adult and older adult females. Butchering tools from burial **207** have been associated with the older female child (see discussion below), while wood, confirmed on only one flint flake, is found with an older male adult. Processing of mineral materials, a more general category of contact material which can be related to other activities such as working hide, with ochre sometimes used as an additive (e.g., [36,37]), was observed only on four flints (Fig 10b). What is interesting is that these four flakes were found only in female burials, with an older child and younger and older adults.

Few patterns can be observed when assessing wear traces by typological category. Flakes, as the largest represented group of artefacts, are mainly either unused (45%, n = 38) or have indeterminate traces (23%, n = 19). Regarding the used flakes, one flake per each of the worked contact materials was observed: meat, butchering, wood and a combination of animal and vegetal activity. Animal material and bone traces have been found on two blades per material category. Four were used for processing mineral materials, while six were used to work hide. Burial **211** stands out for having four flakes used to process hide in different conditions, possibly reflecting different phases of hide work, alongside two flakes for processing mineral materials. Recognised as a multifunctional tool across Stone Age Northern Europe [2,38,39], most of the blades 65% (n = 19) from Zvejnieki burials have no visible traces of use. Two blades were used to cut bone, one to cut hide, and one for processing unidentified animal material. Another two blades have macro traces and two are indeterminate.

Among the formal tools, just under half the scrapers have indeterminate traces and two were likely unused. Four scrapers have macro traces, one was used to process hard animal material and two were possibly employed in hide working. Two of the three knives are fragmented: both have indeterminate traces, while the third from burial **317** has traces associated with processing a hide-like material. The only striking pattern when it comes to assessing the relationship between typology and function are the bifacial points, with the majority (n = 21, 85%) displaying *no visible traces of use*. A further four have indeterminate traces.

**Fig 11:**
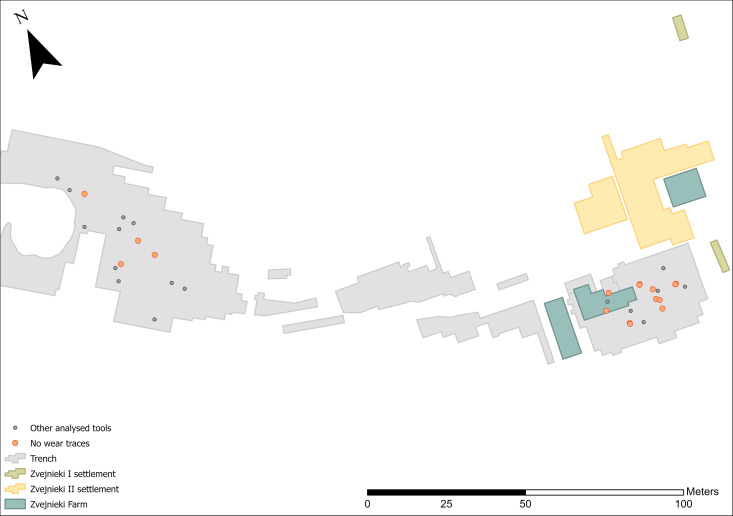
Distribution of lithics in burials with no visible wear (orange) and with wear traces (grey).

No spatial pattern was observed in tools interpreted as utilised. There appears, however, to be a higher frequency of burials containing lithics with no visible signs of use during the later phases of burial activity (Fig 11). Several reasons may account for this pattern. It might reflect a 4th millennium BC tradition of lithics more often being made for the purpose of burial, unused pieces being selected from raw material stores, and/or that wear traces on tools from earlier burials are less well preserved.

### Intra-burial spatial association

For the most part, lithic grave goods from PD have no clear correlation with a specific body part, though this does vary according to type. Scrapers were located near the hands in three cases; one was found near a shoulder and one found near legs, with 7 from unknown locations within the grave. Bifacial points are most strongly (n = 7) associated with the torso, with a further 2 by the arms, 1 by the pelvis, 1 by the shoulder, and 5 with unknown body associations. It is possible that arm, shoulder and pelvis locations represent the binding of bifacial points to upper arms or attachment to belts around the waist [2]. It is unlikely that any of these were embedded in the body given their lack of visible wear (see S1 File for an exception). Blades and flakes display no significant association with any specific body part and are best described as randomly distributed within graves. Three flint knives were found associated with three different body parts – head, shoulder and pelvis.

### Temporal distribution patterns

**Fig 12:**
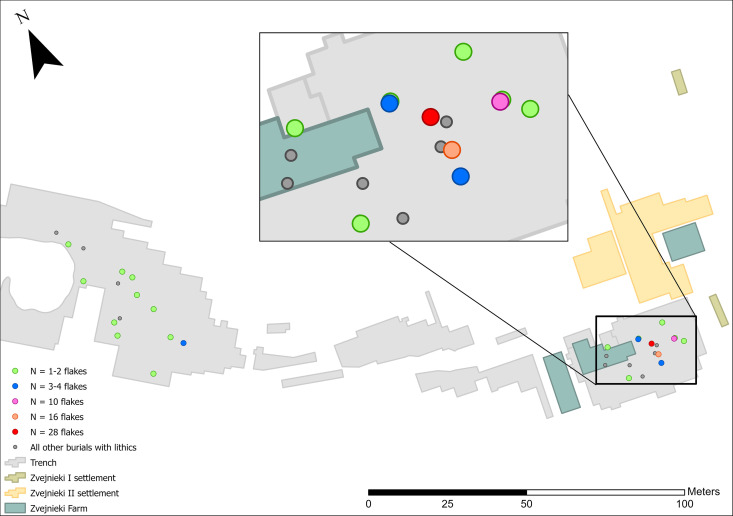
Spatial distribution of flakes (n = 84).

**Fig 13:**
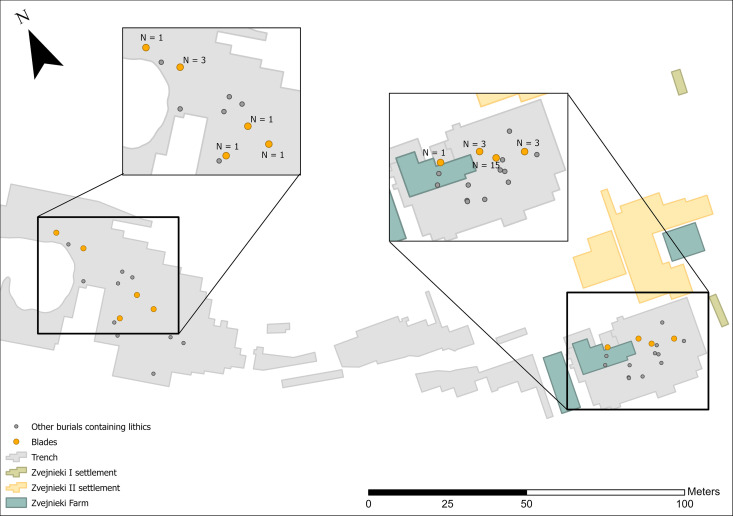
Spatial distribution of blades (n = 29).

**Fig 14:**
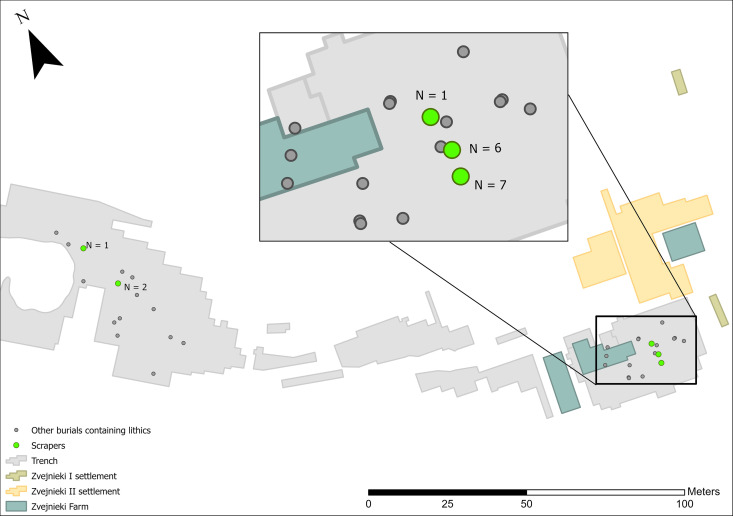
Spatial distribution of scrapers (n = 17).

**Fig 15:**
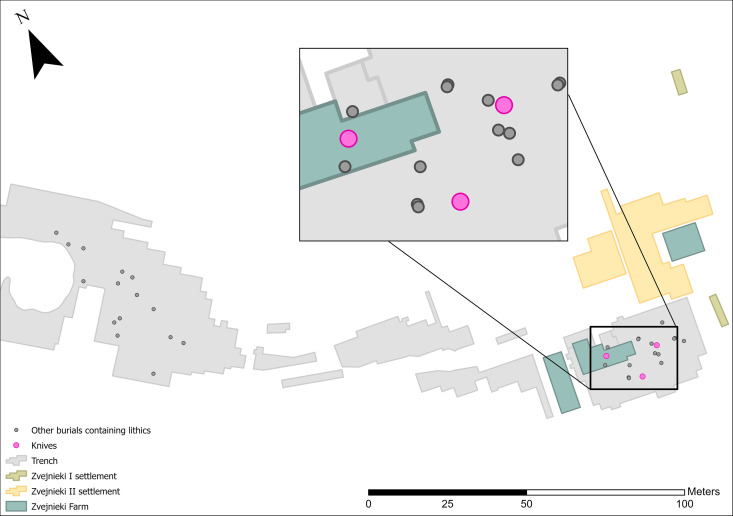
Spatial distribution of knives (n = 3).

**Fig 16:**
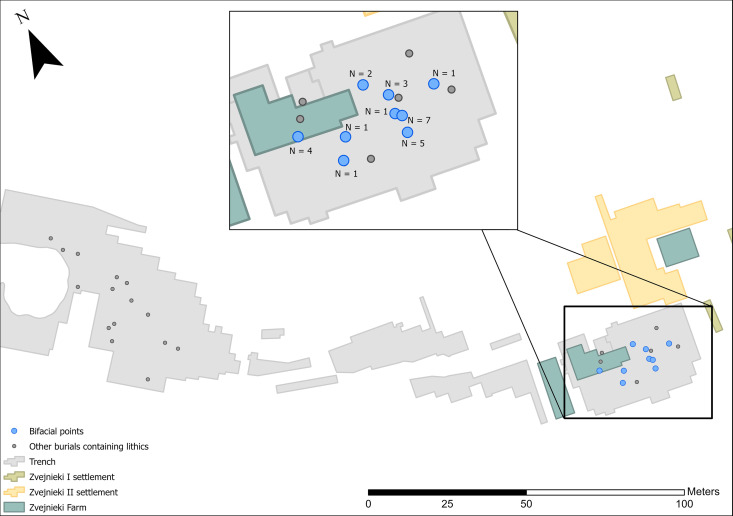
Spatial distribution of bifacial points (n = 25).

The clearest temporal pattern at Zvejnieki is that knapped lithics became more prominent in funerary rites during the 4^th^ millennium BC. In earlier millennia, lithics were rarely included and in very small quantities (1–3 pieces per burial). It is possible that the field record is biased towards the later burials, when more formal tool types were being deposited (Figs 12–16). Excavation methods of the time that prioritised more “exotic” or distinctive items may have led to earlier and less easily recognised (or valued) lithics being overlooked, given these were rarely (except scrapers) being made at that time. In spite of this, flakes (Fig 12) and blades (Fig 13) are found in higher concentrations in later burials in the SE of the site, compared to the earlier burials.

Flakes come from 4^th^ to 7^th^ millennium BC and unknown contexts, but contexts containing 10 flakes or more were all found in the SE part of the cemetery, where burial **211** contains over 30% of the total found across the site.

Blades come from 4^th^, 6^th^–7^th^ millennium BC and unknown contexts; burial, **211** contains over half of all blades.

Of the more formal tool types, scrapers have the broadest temporal and spatial distribution, occurring in 6th and 4th millennium BC, as well as contexts of unknown date (Fig 14). Scrapers are, without doubt, most highly concentrated in burials **201** and **207** located in the SE.

Scrapers derive from 4^th^ and 6^th^ millennium BC and undated contexts, while two burials (**201** and **207**) contain over 75% of the total number.

Knives are uncommon at Zvejnieki. All three that were discovered are from the 4th millennium BC and thus plot spatially to the SE part of the site (Fig 15).

Knives are found only in 4^th^ millennium BC contexts.

Bifacial points are the most common formal tool type found in burials at Zvejnieki, and are exclusively found in graves dating to the 4th millennium BC, located in the southeastern part of the site, with the highest concentrations in burials **201**, **207** and 211 (Fig 16).

Bifacial points are found in 4^th^ millennium BC contexts. Over 60% come from just three burials (201, 207 and 211).

### High density lithic burials

#### Overview of high density lithic burials.

One clear trend in the data is the clustering of 4th millennium BC burials with higher quantities of lithic grave goods in the SE part of the site. This concentration of offerings in just a few burials is a pattern also observed at other Northern and Northeastern European hunter-gatherer cemeteries [40]. Two thirds of the lithic-bearing burials at Zvejnieki contain only one or two items, and while the mean value is 4.8, the median remains at 1. Thus, we define high density burials as those containing more than the average – five or more – flaked lithic artefacts.

**Fig 17:**
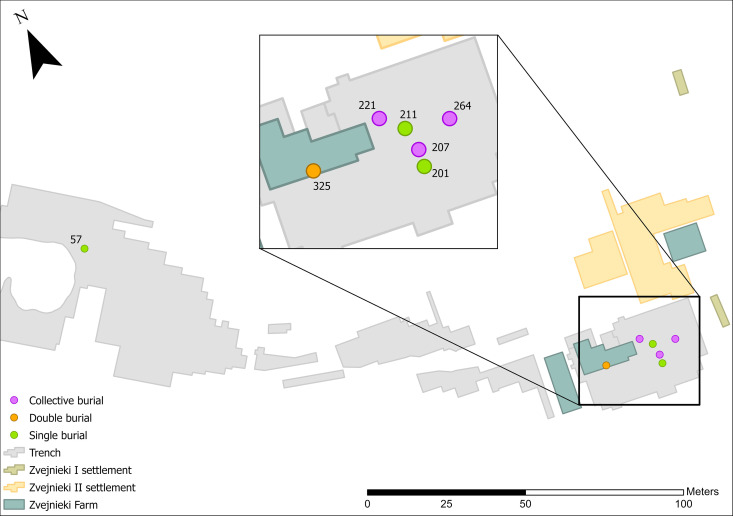
High density lithic burials and other burials with lithic assemblages of interest.

Five individuals (burials **201, 207, 211, 221, and 264**) received the highest quantities of lithic grave goods (Fig 17). As a group, they all fall into the 4th millennium BC date range and show no specific pattern of association between lithics and age categories. In fact, high density lithic burials can best be described as belonging to a cross-section of the buried population. Three of the five burials are female, one is male, one is “unknown”: reflecting a site-wide trend where females are at least, if not more, likely to receive lithic offerings. All modes of burial – single, double and collective inhumations are also represented. Where burials are double or collective, caution is needed when interpreting the associations between lithics and a specific individual. For the following interpretations, the spatial connection of lithics and individuals has been taken from Zagorskis [5] and unpublished excavation reports.

Also included here are two additional burials – **57** (female) and **325** (male) (see Fig 17), which have less than five pieces, but are of special interest because of their chronology and burial practices (burial **57**) and the exclusive offering of four of one type of lithic grave good (burial **325**).

#### Burial 201 (single burial).

An older child, dating to the 4th millennium BC (tooth, reservoir corrected 3650–3000 cal. BC; [12]; skeletal remains of indeterminate sex include the skull, pelvis and forearms. Due to disturbance, no spatial associations between the skeletal remains and grave inventory could be made, yet this older child had one of the richer assemblages of lithic grave offerings (Table 12), including seven scrapers. Two of these are endscrapers with indeterminate wear traces; of the other five scrapers, four display traces of wear associated with working hide, a soft material and a hard animal material, and one displays non-diagnostic traces. As noted elsewhere, none of the five bifacial points displayed wear traces, suggesting they went into the grave unused – possibly made specifically for the burial.

**Table 12:** Quantity and type of lithics found associated with Burial 201.

Tool type	Raw material	Deposit type	Quantity
Scraper	Flint	Primary	7
Bifacial point	Flint	Primary	5
Flake	Flint	Primary	4

#### Burial 207 (collective burial 206–209).

207 belongs to a collective burial (**206–209**). Burials **206** and **207** are older children; **208** and **209** are adult males. Radiocarbon dates are available for individuals **206, 207** and **208** (all human bone). Reservoir corrected age for individual **208** is 4200–3600 cal. BC [12], while the uncorrected ages for burials **207** and **206** are 4050–3950 cal. BC [26] and 4250–3980 cal. BC [41], respectively. An additional date, regarded as the most reliable, is from a Cervid bone (from burial **208**) of 3780–3390 cal. BC [6].

Several aspects of the treatment of individuals in this grave are worthy of note. An older child (burial **206**), indeterminately sexed, had amber discs in the eye sockets and an amber pendant at the back of the head. A deposit of clay around the head area [7] has been interpreted as a death mask, which are found in association with a small number of later burials at Zvejnieki: **206, 225, 263, 275, 276** and **317** [42,43]. The second child, burial **207**, also an older child and genetically sexed as female, had a relatively large collection of flint artefacts, including 16 flakes, 7 bifacial points and 6 scrapers, all in PD or OPD contexts. In fact, **207** is the *only* burial at Zvejnieki with lithics in both PD and OPD contexts. Moreover, this female child has the highest quantity of bifacial points of all excavated individuals at Zvejnieki, and the second highest quantity of scrapers. Most of these come from the OPD “votive deposit” located at her feet. The OPD further contained a high quantity of diverse grave goods made from bone, teeth, tusks, amber, coarse stone etc.

Even though the OPD is described as associated with **207** [7], it is possible that its contents were not intended exclusively for this child. The presence of several flint artefacts in close spatial association with her body does, however, suggest a strong association with lithics. Her immediate burial assemblage includes two bifacial points, neither displaying traces of past use, in keeping with a broader site-wide trend observed for bifacial points, plus two scrapers and several flakes (Table 13). Some have wear traces indicating employment in butchering, meat processing and mineral-related activities. Within the OPD, one flake has been employed in cutting bone, a scraper displays traces from working a medium material, a further three (two scrapers and a bifacial point) had non diagnostic traces. The remaining four bifacial points had no visible traces of wear. The ratio of unused and indeterminate traces is similar between the tools found in direct association the body and the OPD. Elsewhere, an argument has been made that some of the flakes from the OPD are derived from the same nodule [44]. If this is the case, the wear traces visible on some of the lithics indicates different life histories after knapping and prior to deposition in the grave.

**Table 13:** Quantity and type of lithics found associated with Burial 207.

Tool type	Raw materials	Deposit type	Quantity
Flake	Flint	Primary and Other Primary	16
Bifacial point	Flint	Primary and Other Primary	7
Scraper	Flint	Primary and Other Primary	6

#### Burial 211 (single burial).

Burial **211** contains a young adult female, who, based on associated material culture, has been dated to the 4th millennium BC. The axe from this burial has been published previously [1]. Microwear analysis was carried out on these pieces because of the uncertainty surrounding their context (see S1 File).

A 1.5m-0.5m ochre-rich “votive deposit” (OPD) was found in association with burial **211.** Alongside other types of non-lithic grave goods, the deposit includes two complete bifacial points (one apparently broken after excavation and glued back together) and one half of a bifacial point. Again, none of these bifacial points show traces of use. The OPD further contained 28 flint flakes; 15 blades and 1 scraper (Table 14). All the lithics were ochre stained because of the burial environment. Most of this deposit went into the ground unused, or with traces that were poorly developed and not identifiable (see S1 File). The scraper displayed wear traces from hide-working; four other flakes and blades also displayed hide-related wear traces; a blade had animal material traces; and two flakes were likely used to process minerals. The types of contact materials (linked to hide, animal and mineral processing) share similarities to the tools in burial **207**. Like **207** above, it has also been suggested that some of this material may refit to a single nodule [44].

**Table 14:** Quantity and type of lithics found in association with Burial 211.

Tool type	Raw materials	Deposit type	Quantity
Flake	Flint	Other Primary	28
Blade	Flint	Other Primary	15
Bifacial point	Flint	Other Primary	3
Scraper	Flint	Other Primary	1

#### Burial 221 (collective burial 220–225).

Burial **221** is part of a collective burial (**220–225**) containing five adults and a young child. Radiocarbon dates have been published for individuals **221, 224** and **225** (all human bone). The reservoir corrected age for individual **221** is 4050–3500 cal. BC [12] and 4000–3550 cal. BC for individual **225** [33]. First to enter the grave was an adult female, burial **221**; initially sexed as male, but through aDNA analysis [25], has been re-sexed as female. This is significant in that she was furnished with an extensive and varied burial assemblage consisting of numerous amber ornaments, figurines, fishing equipment, worked antler (see [45]), a whetstone and, of key interest to this research, a relatively large flint assemblage (Table 15), including two bifacial points associated with her torso and arm. The lithic assemblage contains similar numbers of fragmented and complete pieces. A flake and two blades were used on indeterminate contact materials of different hardness in scraping/cutting motions. “Mineral” is the only identifiable contact material, visible on a fragmented flake. As mentioned, mineral working traces, though few, are exclusively associated with females at Zvejnieki.

**Table 15:** Quantity and type of lithics found in association with Burial 221.

Tool type	Raw material	Deposit type	Quantity
Blade	Flint	Primary	3
Bifacial point	Flint	Primary	2
Flake	Flint	Primary	2

#### Burial 264 (collective burial 263, 264, 264a).

**Fig 18:**
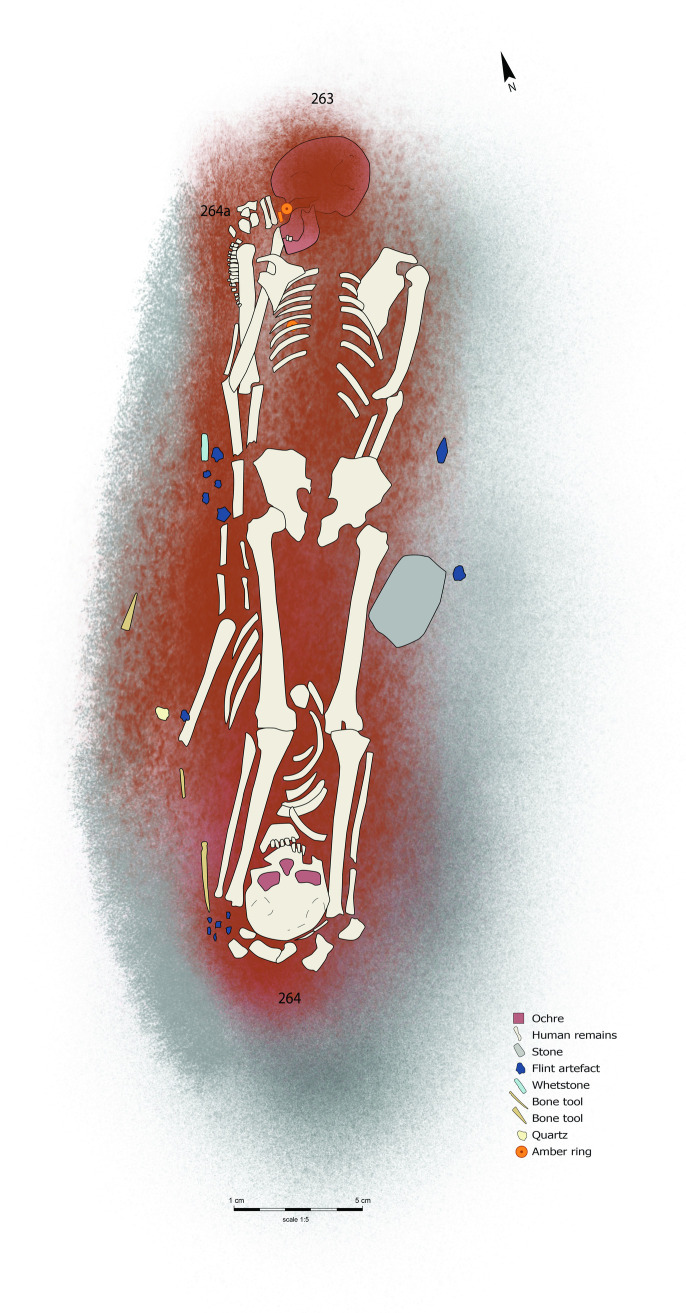
Reconstruction drawing of collective burial 263, 264 and 264a.

Burial **264** is a male older adult, buried in an opposite direction to another male older adult (**263**) buried in a prone position. Just visible at the head area of individual **263**, is younger child **264a**, who is contemporary with both **263** and **264** (Fig 18). Based on the material culture, this collective burial has been dated to the 4th millennium BC.

Drawing by Hege Vatnaland based on the original drawing by Baiba Vaska stored at the Repository of Archaeological Material, Institute of Latvian History.

The infant burial (**264a**) had no associated grave goods. Around the head area of burial **263** was a clay/ochre mixture and two amber discs [7], interpreted as a death mask [42,43]. At the other end of the grave, burial **264** has a relatively large assemblage (Table 16) of tertiary flint flakes and blades; most are complete, just one flake has retouch. All were found in association with the head and leg areas, as was a single quartz flake, unworked, with undiagnostic wear traces. A whetstone was also found nearby, alongside the worked bone objects, while an amber disc was located between the individuals [7]. Only two flakes display diagnostic traces of use – processing bone and hide, while six artefacts bear no visible traces of use, and the rest (n = 5) were interpreted as indeterminate.

**Table 16:** Quantity and type of lithics found in association with Burial 264.

Tool type	Raw material	Deposit type	Quantity
Flake	Flint, quartz	Primary	10
Blade	Flint	Primary	3

#### Burial 57 (single burial) and Burial 325 (double burial 323, 325).

Although they fall slightly short of the limit set for “high density”, burials **57** and **325** both have unique aspects that warrant further discussion.

Burial **57** is an adult female, in a supine position in a grave with a stone setting. She was given four flaked lithic artefacts. Three blades were located by her leg/knees area, two of them broken. The only complete blade was used to cut bone. A scraper, with no visible traces of wear, had been placed by her hand, an arrangement seen in another burial (**82**), where two scrapers are found next to the fingers of the right hand. Several other grave goods, including a zoomorphic staff and unperforated animal teeth were also recovered from this burial [9]. An axe, with hide and mineral working traces on its lower surface indicating its use in an unconventional way, possibly as part of the funerary rites, was contained within a pouch placed by her head (see [1]). Combined, the stone setting, special treatment of the axe, and a diverse burial assemblage, including stone tools, suggests she may have carried some importance within her community. What is perhaps most unusual is that this individual dates to the 6th millennium BC, with a skull fragment returning a date, reservoir-corrected, of 5750−5250 cal. BC [33]. This female thus represents the only example of a pre- 4^th^ millennium BC burial with a relatively high number of flaked lithic artefacts (see Fig 17).

Burial **325** belongs to an adult male and has an uncorrected radiocarbon age of 4230–3960 cal. BC [31]. It belongs to the 4th millennium BC group of burials with numerous bifacial points. What is unusual is that in addition to non-lithic grave goods (Comb Ware pottery fragments), the only lithic offerings in burial **325** were four bifacial points, all of which had been placed on the torso area. Only one is complete, the others are fragmented. While all were patinated, none displayed any visible traces of wear, even at a macro-level. It is possible that these four points were made, and in the case of three points, possibly intentionally broken as part of this individual’s funerary rites.

## Discussion

How do our analyses help us to understand the significance of lithic artefacts in graves at the Zvejnieki cemetery? In terms of general trends, nearly all formal flint artefacts (bar scrapers) are associated with the 4th millennium BC and concentrated in the SE part of the site. This conforms to a trend seen across north-eastern Europe, particularly for bifacial points [14,34]. The 4^th^ millennium also sees higher concentrations of flakes and blades deposited in PD or OPD in the SE area. While selective recovery or excavator bias may be a minor factor, the trend towards the deposition of a wider range of lithic forms over time does seem to be genuine.

The appearance of more formal lithic artefacts in Zvejnieki burials coincides with a major cultural and demographic change in the eastern Baltic Sea area in the beginning of the 4th millennium BC (e.g., [46], for aDNA [47]). Part of this transformation is the spread of new, visually captivating burial customs, including collective burials, clay masks, and the use of red ochre [13,42,43,48,49]. One salient feature of the new burial ritual is the inclusion of visually distinctive objects, often made of various mineral materials, many of which were of non-local origin [44,50]. This is evident at Zvejnieki, where amber, for example, appears in graves, alongside imported flint. Thus, the inclusion of more knapped lithics in burials at Zvejnieki is not so much a local feature, but part of a broader socio-cultural change of attitudes and practices throughout boreal north-east Europe.

Our study also shows that many flaked lithic artefacts entered the grave unused or with traces indicating very brief use-lives. Flakes, blades, and scrapers were more frequently used prior to deposition compared to bifacial points. Scrapers were used on materials of varying hardness. Hide is the most common contact material worked. No strong wear trace pattern could be seen in relation to any other typology, except for the lack of wear traces on bifacial points. More generally, traces are poorly preserved due to taphonomic factors, resulting in post-depositional surface modification. As such, while it was possible to sometimes identify archaeological wear traces, many were classified indeterminate. Some of the more poorly developed wear traces may not have been preserved.

Across all burials, tools used to work different animal-related materials (bone, meat, hide etc.) make up the largest percentage of pieces with preserved wear traces. It may be logical to assume that animal processing activities were part of everyday life for the hunter-fisher-gatherer inhabitants of Zvejnieki. It appears, however, that animal processing tools were also regarded as an appropriate gift for the dead. Used tools, including those for hide and mineral-working, form PD associated with the body and OPD “votive deposits” in small pits, located within and beside the grave. Hide traces feature most frequently in the OPD of the high density lithic burial **211**, which also contains more generic animal traces possibly linked to animal/hide-processing. Hide-working traces previously identified on the axe from burial **57** [1] similarly suggest that the working of this material may have had particular significance, or even been a feature of the funerary rites practised here. Microwear analysis of well-preserved lithics from secure Zvejnieki settlement contexts is now needed to test how far these patterns are repeated amongst the living, and how prevalent animal processing tools are more generally. Bifacial points were more likely to be deposited unused, and frequently broken, indicating that these lithic objects had different biographies. Intentional breakage of bifacial points was recognised by the original excavators [7]. Elsewhere, deposits of just the tip or half the object, have been observed in Finnish burials of a similar date [49] (see also [2]): suggesting that the making and breaking of bifacial points as part of mourning rituals may have played a part in a wider pattern of mortuary behaviour throughout the eastern Baltic region during the 4th millennium BC.

Questions of possible links between lithic artefacts and social identity remain, to some extent open-ended. Many of the burials with the most numerous lithic finds at Zvejnieki are female and/or people of a young age (younger child, older child, younger adult). This effectively challenges persistent “man equals tool” narratives within Stone Age mortuary studies [40,51,52] (see [3] for a critique). At Zvejnieki, issues of gender bias may also have skewed the dataset, with certain lithic objects, particularly the bifacial points, described as ‘a characteristic form of male grave goods although they also occur in the graves of children and adolescents’ [7]. Despite the potential for gender bias, our results, incorporating the latest aDNA sexing data, shows that the percentage of female burials with lithics in primary contexts is broadly the same as males (see Table 6).

Some typological variation exists (see Table 10); for example, scrapers are exclusively given to females. Although bifacial points occur in more male burials, they are found in higher concentrations with females, as is also the case with blades. Given that bifacial points (and possibly other lithic items) were seen by the original excavators as characteristically male items, we expect that any future aDNA sexing of Zvejnieki individuals will likely *increase* the present number of recorded females with lithic burial deposits. Alongside previous studies of the axes, which show a strong correlation with females and children at Zvejnieki [1], our findings challenge received wisdoms about gendered roles and suggest that the relationship between artefacts and social identity in Baltic Stone Age society was more complex than has often been assumed.

We observed only four tools with mineral-working traces. These were found exclusively in female graves. While this is interesting, the number is small, making it difficult to draw any firm conclusions about the relationship between gender and mineral working. The inclusion of tools used to scrape ochre into burials is known from other Stone Age sites [52], with “funerary rite tools” a recently recognised type of Mesolithic grave good [2]. At Zvejnieki, where ochre is a major part of the funerary process [13], mineral-working tools might also have been connected to the ochring of graves.

Counter to what might be expected regarding age associations, lithic deposits are most frequently associated with younger children and older adults (see Table 8). Hide-working tools are found relatively equally in the graves of children and adults. Similarities exist between the lithic assemblages of aDNA sexed female child burial **207** and unsexed child burial **201**. Both assemblages contained utilised tools; some had been used in animal processing activities, including hide working. Different reasons may account for this. Associations with animal processing tools may reflect children’s involvement in butchery and/hide work during the 4th millennium BC. Other possibilities include tools being used by adults for animal and hide-working tasks, with these then deemed suitable funerary offerings for children.

Lithic assemblages from burials **207** and **211** also share some similarities. Both are associated with lithics used to work animal materials and have deposits of scrapers and bifacial points. It has been proposed that some of the lithic materials within these respective burials refit [44]. Archaeological wear traces visible on some of the pieces suggest that, even if they are derived from the same nodules, their life histories were more complex than simply being knapped directly into the graves. It is interesting to note that both individuals are females and that they are located adjacent to each other (see Fig 17). Chronologically, it is conceivable that they belong to a contemporary phase of burial activity. However, considering the absolute dates, temporal connections remain tentative.

Bodily associations are difficult to assess due to the poor spatial information available, though there are hints of a preference for placing scrapers in or near hands and bifacial points on or near the torso. Associations between lithic forms and particular parts of the body, in particular the hands and torso, have been detected elsewhere [2]. Unfortunately, burials of comparable date which share similarities in the deposition of bifacial points in the eastern Baltic area (mostly Finland), rarely have any skeletal remains preserved [44]. Thus, the link between the human bodily form and particular typologies remains an area for future investigation.

## Conclusion

Historic burial archives like Zvejnieki present many challenges. A long and varied history of excavation, with all the shifts of perspective and priority that this implies, means that the data are varied in their resolution. Crucially, it is not yet possible to undertake a substantive comparative analysis of lithic assemblages from the cemetery in relation to those from the adjacent settlement. This must be a critical focus for future work. Similarly, although it was beyond the remit of this study, future comparative research, assessing the relationship between lithic grave goods and the published records relating to the numerous personal adornments and other types of grave goods from Zvejnieki burials, is recommended.

Our study has demonstrated the value in applying a multiproxy approach to all lithic artefacts from primary Stone Age burial contexts. Even with archival burial datasets, valuable insights can be gained into the diverse roles lithics played within Stone Age funerary customs. Future excavations of hunter-gatherer burial sites would benefit from considering the application of this integrated methodology from the outset, including geology, technology, microwear and archaeological context studies. Excavation methods should ensure full recovery of lithics, including geolocation data, while mitigating against surface damage that can be caused by sieving. Similarly, close attention needs to be paid to lithic storage and conservation post excavation to enable functional analyses.

Finally, results from our research on the flaked lithic assemblages from the Zvejnieki cemetery, alongside a previous study of the stone axes [1], challenge long-held assumptions about the strictly “utilitarian” nature of lithics within Stone Age communities. Stone tools, crucial to everyday tasks, were more than mundane utensils; they played a key part in people’s mourning rituals. At times, it appears it was necessary to make, use and break lithics as part of funerary rites. Their relatively equal placement into the graves of both sexes and all age groups (with a female given the most flaked lithic grave goods) clearly establishes the shared significance of stone tools across *all* sectors of Zvejnieki society – at least, at the graveside. To date, Stone Age funerary studies have tended to overlook the numerous lithic artefacts deposited within grave contexts [2,3]. Our study underscores how important lithics are to broadening understandings of hunter-gatherer attitudes to death, identity and commemoration.

## Supporting information

S1 File Supporting Information for the Stone Dead database. (DOCX)

S2 File Inclusivity in Global Research. (DOCX)
