# Supplementary material for: Multiproxy study reveals equality in the deposition of flaked lithic grave goods from the Baltic Stone Age cemetery Zvejnieki (Latvia)
Source: PLoS One. 2025 Sep 10;20(9):e0330623. doi: 10.1371/journal.pone.0330623 (PMC12422477; doi:10.1371/journal.pone.0330623)
Supplement: S1 File — (DOCX) [file pone.0330623.s001.docx]

# **S1 Supporting Information for the Stone Dead database**

### Stone Dead Project database overview

Details for the categories used in the Stone Dead Project database are set out below. Where needed, a brief rationale is provided for why certain decisions have been made. The database [1] is fully open access and is hosted by the Archaeological Data Service, York, UK.

In columns A-F information on the **archaeological context** is provided, including the numbering systems used, burial and deposit types, and dating classifications. In columns G-I can be found information on the human remains, such as sex, age category and body parts associated with lithics. Details on the **lithic grave goods** (raw materials, technology, measurements, lithic surface condition, microwear traces) can be found in columns J-Z. Columns AA-AB provide basic information on the presence of **other grave offerings**, including other types of grave goods and ochre. Finally, columns AC-AD contain the X and Y spatial coordinates used to plot each burial.

As mentioned in the main text, a total of **330** graves have been excavated so far, with at least **350** individuals recorded [2,3], (**Fig 1b**). Out of this number, based on the two main publications on the Zvejnieki cemetery [4,5], and our new observations, we associated 146 individuals with any kind of artefacts. This total represents all individuals found, including those from disturbed burials, and objects interpreted as coming from a secondary context. However, a recent study that focused on bone tools [3] and analysed only burials with a secure context concluded the total number of individuals with grave goods as 115.

### Archaeological contexts

#### Numbering

Many of the lithics were given the same museum accession number, e.g. several pieces from burials 211 and 207, for this reason we have allocated our own *Sample Numbers* (Column A) as unique identifiers. Some of the finds from recent excavations [5] have not been given museum accession numbers; we have used NN (No Number) in these cases. *Museum numbers* (Column B) refer to the accession number given to individual artefacts. A few lithic artefacts from the more recently excavated burials (317, 320 and 325) have no museum number - for these we have assigned ‘NN’. In Column C are the *Burial numbers* assigned during the different excavation campaigns. In Column D, burial type is recorded. S connotes a single burial, D a double burial and C for collective burials containing more than two individuals. All information derived from Zagorskis [4] and Larsson et al. [5] and original fieldwork documentation.

#### Deposits

In Column E is the type of *Deposit* the lithics are associated with. ‘P’ stands for Primary, ‘OPD’ stands for Other Primary Deposit. Lithics from Secondary Deposits have not been included in this spreadsheet, based on context information. Where lithics were recorded as being part of fill, whether it be the black settlement deposits or other types of fills, e.g., grey, gravel etc., these were deemed Secondary rather than Primary. An exceptional lithic find, a trapeze arrowhead embedded in the individual’s thoracic vertebra, was recorded for burial 179, which contained secondary fill deposits [6]. This artefact is not recorded in our primary database.

Burial 57 has issues concerning the description of the deposits. Several flint and quartz artefacts, including an end scraper and part of a bifacial point, as well as the axe, are described as coming from the ‘black earth’, suggesting a secondary deposit [4]. It seems implausible, however, that a complete axe would have been accidentally deposited into a grave. As such, we previously treated this object as an intentional primary deposit [7]. While acknowledging that the other lithics, including formal tool types, may also have formed primary deposits, we have maintained the “black earth/secondary deposit exclusion rule” for the smaller flaked lithic artefacts found in the grave: knowing secondary deposits frequently contain flaked lithic materials.

#### Dating

Column F contains a *Millennia date*, which is an approximate age category. These are based on a) radiocarbon dates of grave goods and human bone with published reservoir correction, and 14-C dates with confirmatory material culture; b) radiocarbon dates of human bone without published reservoir corrections or isotope values (marked with a question mark); c) associated material culture (annotated with an asterix). ‘Unknown’ is used when no chronological identification could be made.

### Human remains

#### Sex

In Column G, Male (*M)* and Female *(F);* Sex identified via DNA is marked with ‘*’. *CI* stands for Child/Indeterminate and *AI* for ‘Adult/Indeterminate’. All children without DNA sexing are CI. For adults, when the original osteoarchaeological identification was “Male?” or “Female?” these have been recorded as “M?” And “F?” respectively.

#### Age

In Column H, we employ the Stone Dead Age Classes made up of five categories: *younger child, older child, younger adult, adult, older adult*. We have used more simplified age ranges from those used in Zagorskis [4], Nilsson Stutz & Larsson [8] and Larsson et al. [5]. Age categories for children as in Zagorskis [4] - Infans I, 0-7 years and Infans II, 7-14 years are recorded as *younger child* and *older child*. Age category of Juvenis 14-17 [4] we mark as *younger adult*. Age group of adults (20-40) as in Zagorskis [4] and 25-40 as used in Larsson et al. [5] are recorded here as *adult*. Both maturus (40-60) and senilis [4] are marked as *older adult* in our database.

#### Skeletal element spatial association

Column I contains information on the spatial association between the skeletal remains and the lithic artefact, which is based on data from field and museum documentation and Zagorskis [4]. ‘Unknown’ is used when it is not clear which part of the body the lithic was associated with. The association is usually made with one specific body part, except for burial 264. In this case the primary deposit was detailed as located near to the head and leg - ‘thin flakes of blueish flint at left femur and head’ [4].

### Lithic grave goods

#### Lithic raw materials

Column J denotes the determination of lithic *raw material* based on naked eye only. Broad geological classifications ‘flint’, ‘quartz’, and ‘slate’ were used. ‘Quartz’ denotes a common crystalline form of the silica mineral, while ‘slate’ is a general term for fine-grained, metamorphic foliated rocks. ‘Flint’ is broadly understood here as a cryptocrystalline form of quartz found in limestone or chalk. Column K provides detail on the *colour* of raw material; however, this was not used in determining more accurate geological provenance (of flint) due to discoloration and patina on the objects.

#### Technology

Information on *typology* is provided in Column L using classifications that best capture what the object is. This means that artefacts with no secondary working (e.g unretouched flakes) are listed in the same way as lithics with secondary modification (e.g. bifacial points). Columns M-O detail the completeness, reduction sequence and presence of secondary working on each piece.

#### Measurements

Maximum length, width and thickness measurements are captured in Columns P-R. Due to a change in decision regarding recording lithics under 20mm, there is an absence of thickness measurement information for a total of four lithics.

#### Condition

The surface condition of lithics is recorded under four categories: *post-depositional surface modification (PDSM*) and *PDSM appearance* (Columns S-T), *patination* (Column U), *thermal stress* (Column V) and ochre stained lithics (Column W). PDSM, patination, thermal stress and ochre staining are recorded as present or absent (yes/no). *PDSM appearance* specifies the nature of surface modification observed on each piece.

#### Microwear

Presence/absence of *microwear traces* is recorded in Column X and the *motion* in which the tool was used (based on the directionality of traces) is recorded in Column Y. Possible types of *contact material* worked are listed in Column Z. Where possible, a specific material and its condition is provided (e.g., fresh hide). Where this level of interpretation was not possible, the relative hardness (e.g., hard, medium, soft) is given. In Northern European Stone Age contexts, soft materials typically include plants and fresh hide, medium materials might include some woods, dry hides and clay; harder materials include osseous materials, harder woods and minerals such as amber and stones. At times, the hardness and a broad material classification was possible - for example, hard animal material. Some samples were defined as non-diagnostic. This means that the lithic tool was most likely used, but the traces have either been covered or cancelled by PDSM. As the quantity of preserved traces or heaviness of PDSM enabled us to provide a more specific interpretation these samples are listed as non-diagnostic.

### Other grave offerings

#### Ochre and other grave goods

Whether *ochre* is present in the burial has been recorded in Column AA as yes/no. Ochre information is based on appendices in Zagorskis [4]. The presence/absence of *other grave goods* (excluding flaked lithics) is recorded as yes/no in Column AB. This mostly includes objects made from animal materials, predominantly teeth, with the addition of mineral artefacts, for example, amber and coarse stone [3,4].

#### References

1. Little A, Petrović A, Bates J, Macāne A, Nordqvist K. Stone Dead: Lithics Database for the Zvejnieki Burial Ground, Latvia, 2021–2023 [dataset]. York: Archaeology Data Service; 2025. <https://doi.org/10.5284/1132983>
2. Zagorskis F. Zvejnieku akmens laikmeta kapulauks. Riga: Zinātne; 1987.
3. Macāne A. Stone Age companions: humans and animals in hunter-gatherer burials in North-Eastern Europe [dissertation]. Gothenburg: University of Gothenburg; 2022. (GOTARC, Series B, Gothenburg Archaeological Theses; vol. 81).
4. Zagorskis F. Zvejnieki (Northern Latvia): Stone Age cemetery. BAR Int Ser 1292. 2004.
5. Larsson L, Nilsson Stutz L, Zagorska I, Bērziņš V, Ceriņa A. New aspects of the Mesolithic-Neolithic cemeteries and settlement at Zvejnieki, Northern Latvia. Acta Archaeol. 2017; 88:57–93. <https://doi.org/10.1111/j.1600-0390.2017.12177.x>
6. Meadows J, Bērziņš V, Brinker U, Lübke H, Schmölcke U, Staude A, et al. Dietary freshwater reservoir effects and the radiocarbon ages of prehistoric human bones from Zvejnieki, Latvia. J Archaeol Sci Rep. 2016; 6:678–89. doi:10.1016/j.jasrep.2015.10.024
7. Petrović A, Macāne A, Strautnieks I, Kalniņa L, Holmqvist E, Hunter EM. Et. al. Stone axes throw new light on Baltic stone age mortuary rites. Sci Rep. 2024; 14:16219. doi:10.1038/s41598-024-66854-9
8. Nilsson Stutz L, Larsson L. Disturbing the dead: archaeothanatological analysis of the Stone Age burials at Zvejnieki, Latvia (excavated 2006–2009). J Archaeol Sci Rep. 2016; 10:715–24.
